# Characterization in nonhuman primates of (*R*)-[^18^F]OF-Me-NB1 and (*S*)-[^18^F]OF-Me-NB1 for imaging the GluN2B subunits of the NMDA receptor

**DOI:** 10.1007/s00259-022-05698-9

**Published:** 2022-02-02

**Authors:** MingQiang Zheng, Hazem Ahmed, Kelly Smart, Yuping Xu, Daniel Holden, Michael Kapinos, Zachary Felchner, Achi Haider, Gilles Tamagnan, Richard E. Carson, Yiyun Huang, Simon M. Ametamey

**Affiliations:** 1 PET Center, Yale University, New Haven, CT USA; 2 Institute of Pharmaceutical Sciences, ETH Zurich, Zurich, Switzerland; 3 Jiangsu Institute of Nuclear Medicine, Wuxi, Jiangsu, China

**Keywords:** [^18^F]OF-Me-NB1, GluN2B subunit, NMDA receptor, PET imaging, Nonhuman primates

## Abstract

**Purpose:**

GluN2B containing *N*-methyl-*D*-aspartate receptors (NMDARs) play an essential role in neurotransmission and are a potential treatment target for multiple neurological and neurodegenerative diseases, including stroke, Alzheimer’s disease, and Parkinson’s disease. (*R*)-[^18^F]OF-Me-NB1 was reported to be more specific and selective than (*S*)-[^18^F]OF-Me-NB1 for the GluN2B subunits of the NMDAR based on their binding affinity to GluN2B and sigma-1 receptors. Here we report a comprehensive evaluation of (*R*)-[^18^F]OF-Me-NB1 and (*S*)-[^18^F]OF–Me-NB1 in nonhuman primates.

**Methods:**

The radiosynthesis of (*R*)-[^18^F]OF-Me-NB1 and (*S*)-[^18^F]OF-Me-NB1 started from ^18^F-fluorination of the boronic ester precursor, followed by removal of the acetyl protecting group. PET scans in two rhesus monkeys were conducted on the Focus 220 scanner. Blocking studies were performed after treatment of the animals with the GluN2B antagonist Co101,244 or the sigma-1 receptor antagonist FTC-146. One-tissue compartment (1TC) model and multilinear analysis-1 (MA1) method with arterial input function were used to obtain the regional volume of distribution (*V*_T_, mL/cm^3^). Occupancy values by the two blockers were obtained by the Lassen plot. Regional non-displaceable binding potential (*BP*_ND_) was calculated from the corresponding baseline *V*_T_ and the *V*_ND_ derived from the occupancy plot of the Co101,244 blocking scans.

**Results:**

(*R*)- and (*S*)-[^18^F]OF-Me-NB1 were produced in > 99% radiochemical and enantiomeric purity, with molar activity of 224.22 ± 161.69 MBq/nmol at the end of synthesis (*n* = 10). Metabolism was moderate, with ~ 30% parent compound remaining for (*R*)-[^18^F]OF-Me-NB1 and 20% for (*S*)-[^18^F]OF-Me-NB1 at 30 min postinjection. Plasma free fraction was 1–2%. In brain regions, both (*R*)- and (*S*)-[^18^F]OF-Me-NB1 displayed fast uptake with slower clearance for the (*R*)- than (*S*)-enantiomer. For (*R*)-[^18^F]OF-Me-NB1, both the 1TC model and MA1 method gave reliable estimates of regional *V*_T_ values, with MA1 *V*_T_ (mL/cm^3^) values ranging from 8.9 in the cerebellum to 12.8 in the cingulate cortex. Blocking with 0.25 mg/kg of Co101,244 greatly reduced the uptake of (*R*)-[^18^F]OF-Me-NB1 across all brain regions, resulting in occupancy of 77% and *V*_ND_ of 6.36, while 0.027 mg/kg of FTC-146 reduced specific binding by 30%. Regional *BP*_ND_, as a measure of specific binding signals, ranged from 0.40 in the cerebellum to 1.01 in the cingulate cortex.

**Conclusions:**

In rhesus monkeys, (*R*)-[^18^F]OF-Me-NB1 exhibited fast kinetics and heterogeneous uptake across brain regions, while the (*S*)-enantiomer displayed a narrower dynamic range of uptake across regions. A Blocking study with a GluN2B antagonist indicated binding specificity. The value of *BP*_ND_ was > 0.5 in most brain regions, suggesting good *in vivo* specific binding signals. Taken together, results from the current study demonstrated the potential of (*R*)-[^18^F]OF-Me-NB1 as a useful radiotracer for imaging the GluN2B receptors.

## Introduction

There have been great research efforts to develop suitable positron emission tomography (PET) imaging agents targeting the *N*-methyl-*D*-aspartate receptor (NMDAR) complex. Radioligands developed to date have targeted mainly the (1) PCP-binding site, (2) glutamate-binding site, (3) glycine-binding site, and (4) ifenprodil-binding site [1–6]. Ligands developed for the ifenprodil-binding site are specific to the GluN2B subunits of NMDAR. This receptor subtype is a potential treatment target for multiple neurological and neurodegenerative diseases, including stroke, Alzheimer’s disease (AD), and Parkinson’s disease (PD) [7–12]. In the adult brain, the GluN2B subunit is mainly expressed in the forebrain, a region that regulates cognitive functions. Protein and mRNA expression levels of GluN2B subunits in hippocampal subregions were significantly reduced during increasing AD neuropathology [13]. In contrast, the GluN2B-containing NMDA receptors increased in the hippocampal CA3 area in the brain of schizophrenia patients [14]. Therefore, GluN2B selective antagonists have been developed as potential therapeutic agents for the treatment of cognitive deficits with an improved safety profile [15]. Active research has also been directed at the development of PET imaging agents to assist in the screening and optimization of drug candidates. However, despite more than 20 years of intensive efforts, no suitable GluN2B PET imaging agents are available for clinical research until recently, with the first-in-human evaluation of (*R*)-[^11^C]Me-NB1 [16]. The primary reasons for the failures are (1) low brain uptake of the radioligands. (2) brain uptake pattern inconsistent with known GluN2B expression; (3) off-target binding *in vivo*, especially to sigma-1 receptor; (4) presence of radioactive metabolite(s) in the brain [17].

**Fig. 1:**
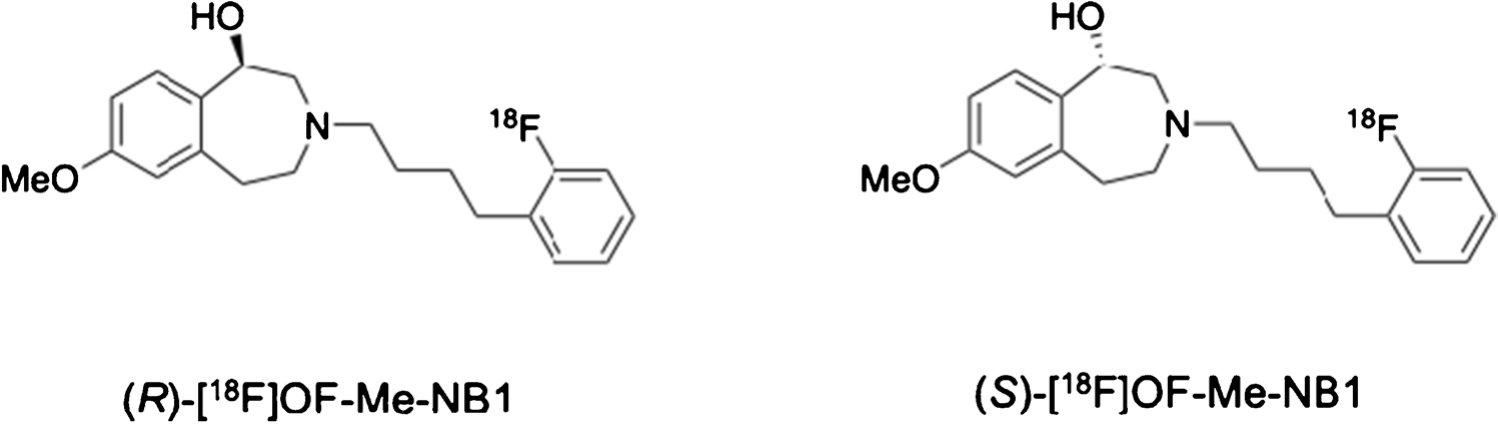
Chemical structures of (*R*) and (*S*)-[^18^F]OF-Me-NB1

Ifenprodil is an inhibitor of the GluN2B subunit-containing NMDARs (*K*_i_: 10 nM) [11]. Modifications of ifenprodil resulted in compounds with preferred binding to either the GluN2B subunit or sigma-1 receptor [18–21]. Our group previously reported [^11^C]Me–NB1, a racemic GluN2B antagonist derived from ifenprodil, with high binding affinity to GluN2B (*K*_i_: 5.4 nM) and selectivity over sigma-1 and sigma-2 receptors (*K*_i_: 182 and 554 nM, respectively) [22]. The (*R*)-enantiomer of [^11^C]Me–NB1 was demonstrated in rodents to have good brain uptake and higher specific binding signals than the (*S*)-enantiomer and has recently been evaluated in humans [16]. Further structural modifications of [^11^C]Me-NB1 led to compounds suitable for labeling with the longer half-life ^18^F-nuclide, and thus the discovery of [^18^F]OF-Me-NB1 [Fig. 1][23].

Similar to [^11^C]Me-NB1, the (*R*)-enantiomer of [^18^F]OF-Me-NB1 was found to have a high binding affinity to GluN2B (*K*_i_: 4 nM) and high selectivity over the sigma-1 receptor (*K*_i_: 100 nM). Both *in vitro *and *in vivo *studies in rodents indicated (*R*)-[^18^F]OF-Me-NB1 as a promising radiofluorinated PET tracer for imaging the GluN2B subunit-containing NMDARs[23]. In the current study, we investigated (*S*)-[^18^F]OF-Me-NB1 and (*R*)-[^18^F]OF-Me-NB1 in nonhuman primates.

## Materials and methods

### Chemistry

Synthesis of the racemic and enantiopure (*R*)- and (*S*)-OF-Me-NB1, and their corresponding aryl boronic esters as precursors followed the previously reported procedures [23]. The absolute configuration of (*S*)-(−)-OF-Me-NB1 and (*R*)-(+)-OF-Me-NB1 were confirmed by circular dichroism.

### Radiochemistry

H_2_^18^O was obtained from Huayi Isotopes (Toronto, Canada). Anion exchange Chromafix cartridges (PS–HCO_3_) were purchased from Macherey–Nagel (Dueringen, Germany). Solid-phase extraction (SPE) cartridges were purchased from Waters Corporation (Milford, MA, USA). The HPLC system used for purification of the tracers included a Shimadzu LC-20A pump, a Knauer K200 UV detector, and a Bioscan *γ*-flow detector, with a semi-preparative HPLC column (Agilent XDB, 9.4 × 250 mm, 5 μm; mobile phase: 27/73 acetonitrile/0.1 M ammonium formate with 5% acetic acid, pH 4.2; flow rate; 5 mL/min). The HPLC system used for quality control analysis included a Shimadzu LC-20A pump, a Shimadzu SPD-M20A PDA, or SPD-20AUV detector (wavelength set at 230 nm), a Bioscan *γ*-flow detector, with a Luna C18(2) column (5 μm, 4.6 × 250 mm) eluting with 36/64 acetonitrile/0.1 M ammonium formate with 5% acetic acid, pH 4.2, at a flow rate of 2 mL/min. The enantiomeric purity was determined by chiral HPLC with a Daicel CHIRALPakIA (5 μm, 4.6 × 250 mm) eluting with 90/10 hexane/ethanol at a flow rate of 1 mL/min.

[^18^F]Fluoride was produced via the ^18^O(p.n)^18^F nuclear reaction in a 16.5 MeV GE PETtrace cyclotron (Uppsala, Sweden). The cyclotron produced aqueous [^18^F]fluoride solution in [^18^O]water was trapped on the anionic exchange resin cartridge, then eluted off with a solution of Kryptofix 222 (6.3 mg/mL in acetonitrile), K_2_C_2_O_4_ (1.0 mg/mL in water), and K_2_CO_3_ (0.1 mg/mL in water) to a 5 mL borosilicate glass reaction vial. The elution solution was evaporated at 110 °C for about 5 min under a nitrogen stream, followed by azeotropic drying with two sequential additions of 0.4 mL acetonitrile. After cooling, 20 mL of air was pushed to the reaction vial, followed by a solution of the boronic ester precursor (6 mg) and Cu(OTf)_2_(Py)_4_ (12 mg) in anhydrous dimethylacetamide (0.4 mL). The mixture was stirred and heated at 120 °C for 20 min and quenched with acetonitrile/water (1:1, *v/v*, 0.8 mL). A solution of 10 N NaOH (0.4 mL) was then added to the reaction vial, and the mixture was stirred at 90 °C for 15 min. After neutralization with 8 N HCl (0.5 mL), the crude reaction mixture was purified by semi-preparative HPLC. The radioactive product peak from 24 to 29 min was collected and diluted with 50 mL of water. The solution was passed through a Waters C18 SepPak cartridge. The SepPak was washed with 1 mM HCl (10 mL) and dried with air. The product was eluted off the SepPak with 1 mL of USP EtOH, followed by 3 mL of USP saline. The combined solution was then passed through a 0.22 μm GV filter (Millipore, Sigma) into a 10 mL dose vial pre-charged with 7 mL of USP saline. For the determination of enantiomeric purity, an aliquot of the EtOH solution was diluted with hexane/ethanol (90/10, *v/v*) and analyzed by chiral HPLC.

### Measurement of lipophilicity (log D_7.4_)

Lipophilicity (log D_7.4_) was measured using the method previously reported [24]. Log D_7.4_ was calculated as the ratio of decay-corrected radioactivity concentrations in 1-octanol and phosphate-buffered saline (PBS, pH = 7.4, Dulbecco). Six consecutive equilibrations were performed until a constant value of log D_7.4_ was obtained.

### PET imaging in rhesus monkeys

A total of seven PET scans in two monkeys were acquired. Each monkey underwent one baseline scan with (*R*)-[^18^F]OF-Me-NB1, and two blocking scans at 10 min after the injection of the GluN2B antagonist Co-101,244 (0.25 mg/kg) or the sigma-1 antagonist FTC-146 (0.027 and 0.125 mg/kg). Co-101,244 and FTC-146 were previously used by Cai et al. to determine the *in vivo* binding specificity and selectivity of a radiotracer from the same scaffold, [^11^C]NR2B-SMe, in rodents [25]. In addition, a baseline scan with (*S*)-[^18^F]OF-Me-NB1 was also performed in one monkey. All procedures were approved by the Yale University Institutional Animal Care and Use Committee.

Animals were sedated with a combination of alfaxalone (2 mg/kg), midazolam (0.3 mg/kg), and dexmedetomidine (0.01 mg/kg) and maintained in an anesthetized state with 1.5–2.5% isoflurane in oxygen. Heart rate, blood pressure, respiration rate, oxygen saturation, and respiration rate were monitored continuously. An arterial line was placed in the radial artery for blood sampling.

Dynamic PET data were acquired on the Focus 220 scanner (Siemens Medical Solutions, Knoxville, TN, USA). Before radiotracer injection, a 9 min transmission scan was obtained for attenuation correction. The radiotracer (10 mL) was administered intravenously as a slow bolus over 3 min. Emission data were collected in list mode for 90 or 120 min and binned into frames of increasing durations (6 × 30 s, 3 × 1 min, 2 × 2 min, and 16 or 22 × 5 min).

#### **Arterial input function measurement and metabolite analysis**

Measurements of plasma activity and parent fraction over time were performed as previously described [24, 26]. Arterial or venous blood samples were collected at preselected time points and assayed for radioactivity in whole blood and plasma with cross-calibrated gamma counters (Wizard 1480/2480, Perkin Elmer, Waltham, MA, USA). The volume of the whole blood or plasma (50–200 µL) was determined by the weight of the sample. Parent fraction was calculated as the ratio of the total radioactivity in fractions containing the parent compound to the total amount of radioactivity collected and fitted with an inverted gamma function and corrected for filtration efficiency. The arterial plasma input function (AIF) was then calculated as the product of the total counts in the plasma and the interpolated parent fraction at each time point.

#### **Measurement of radiotracer-free fraction (f**_**p**_**) in plasma**

The ultrafiltration method was used for measuring the unbound portion (free fraction) of [^18^F]OF-Me-NB1 in plasma as previously described [24]. The *f*_P_ was determined as the ratio of the radioactivity concentration in the filtrate to the total activity in plasma. Measurements of *f*_P_ were performed in triplicate for each scan.

#### **Image processing**

High-resolution magnetic resonance images were acquired with a Siemens 3 T Trio scanner for the region of interest (ROI) definition [27]. PET emission data were attenuation-corrected using the transmission scan, then reconstructed using a Fourier rebinning and filtered back-projection algorithm. PET images summed from the first 10 min of each scan were registered to the anatomical MR image. Inverted transformations were applied to register an atlas ROI mask to the PET image, and time-activity curves (TACs) were extracted from each ROI: frontal, occipital, temporal, cingulate and insular cortex, caudate, putamen, globus pallidus, nucleus accumbens, amygdala, hippocampus, thalamus, pons, substantia nigra, cerebellum, and centrum semiovale.

#### **Kinetic modeling**

Kinetic parameters were determined by fitting the regional TACs and metabolite-corrected arterial input functions to the one-tissue compartment (1TC) and two-tissue compartment (2TC) models, as well as the multilinear analysis 1 (MA1) method with starting time of 30 min [28]. The regional volume of distribution (*V*_T_, mL/cm^3^) was derived in each case.

For the blocking studies, occupancy plots were constructed using regional *V*_T_ values at baseline and differences in *V*_T_ between baseline and blocking scans. Percent target occupancy across the brain and non-displaceable volume of distribution (*V*_ND_) was then determined from the resulting linear relationship, where the slope is the uniform receptor occupancy by the drug in the brain [29].

## Results

### Chemistry

Enantiomeric pure (*R*)- and (*S*)-OF-Me-NB1 and their boronic ester radiolabeling precursors were prepared in good chemical yields as previously reported [23].

### Radiochemistry

**Fig. 2:**
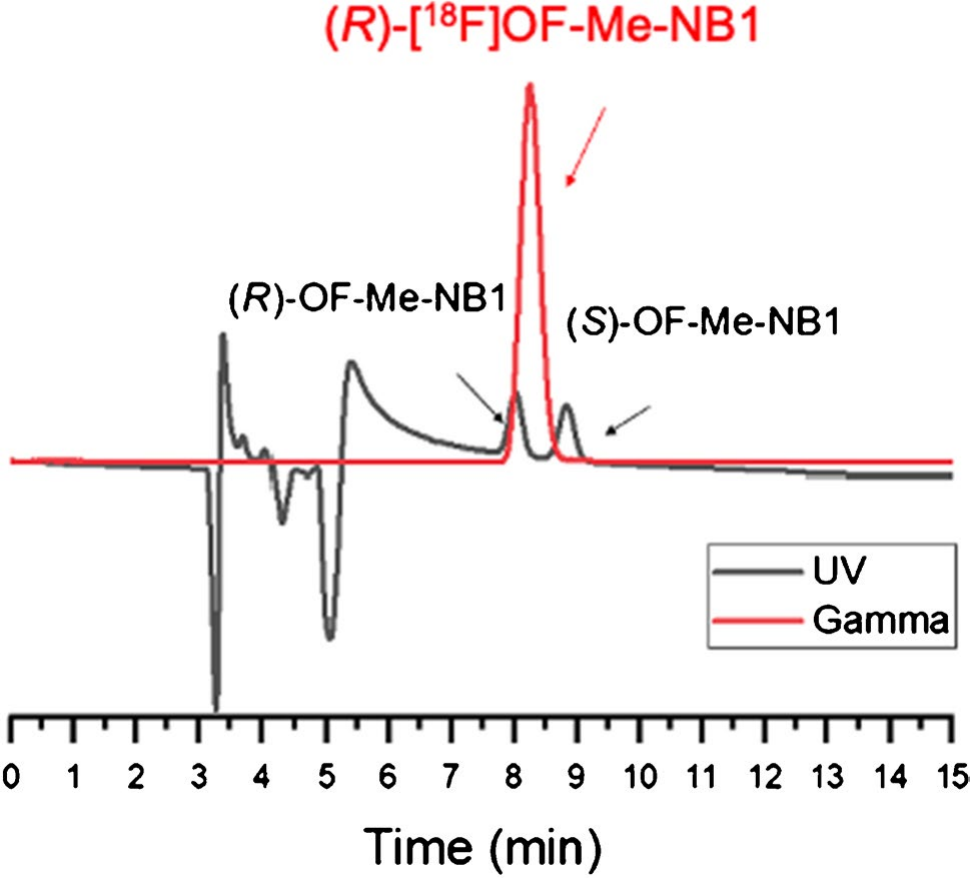
A representative analytical chiral HPLC chromatogram for (*R*)-[^18^F]-OF-Me-NB1(red, gamma), co-injected with the racemic reference standard (black, UV). Column: Daicel CHIRALPak IA, 250 × 4.6 mm, 5 µm. Mobile phase: 90/10 hexane/EtOH. Flow rate: 1 mL/min

Both (*R*)- and (*S*)-[^18^F]OF-Me-NB1 were prepared in 11 ± 3.5% radiochemical yield (*n* = 10, decay-uncorrected). Radiochemical purity was greater than 99%, with molar activity of 224.22 ± 161.69 MBq/nmol at the end of the synthesis (EOS, *n* = 10). Total synthesis time was about 100 min including purification and product formulation. There was no racemization during the radiolabeling and deprotection step. Enantiomeric purity was > 98% for both (*R*)- and (*S*)-[^18^F]OF-Me-NB1, as determined by chiral HPLC analysis (Fig. 2).

### Measurement of log D_7.4_

The measured log D_**7.4**_ value was 2.85 ± 0.01 (*n* = 4) for (*R*)-[^18^F]OF-Me-NB1. This value is within the range of brain-penetrating radioligands.

### PET imaging experiments in rhesus monkeys

#### **Injection Parameters**

The injected activity ranged from 122 to 174 MBq, corresponding to an injection mass of 0.01–0.05 ug/kg. No significant changes in vital signs were observed in the baseline scans. Blood pressure dropped by ~ 20% after the injections of blocking drug and tracer for the blocking scan with either Co-101,244 or FTC-146.

#### **Plasma Analysis**

**Fig. 3:**
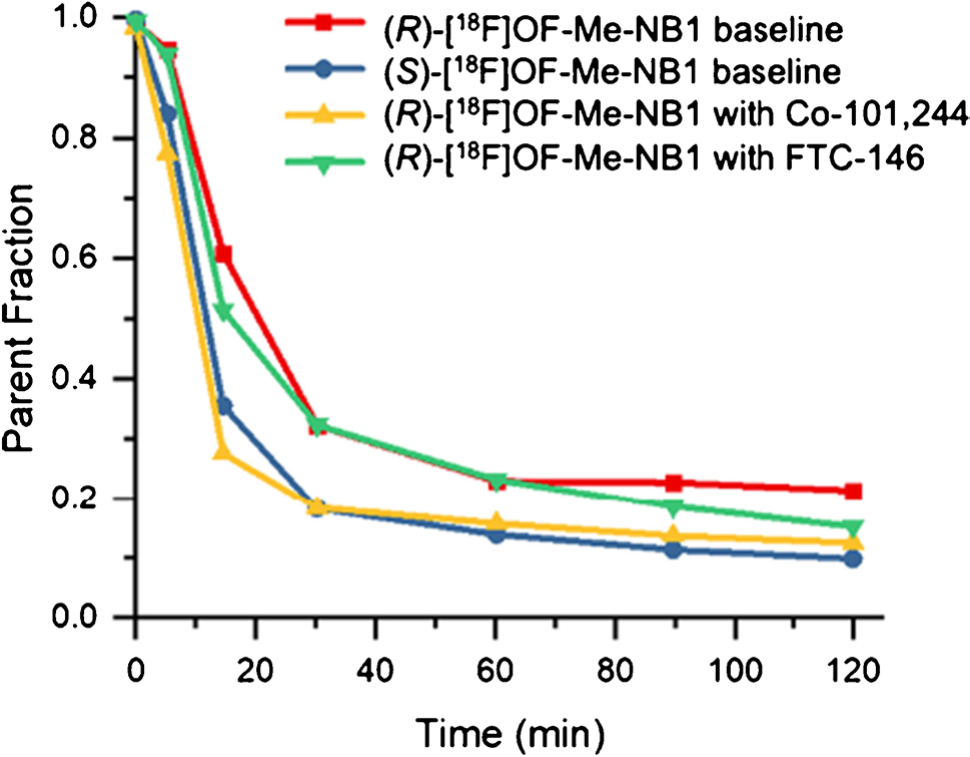
Parent fraction of (*R*)- and (*S*)-[^18^F]OF-Me-NB1 over time in the same monkey

Figure 3 shows the parent fractions of (*R*)- and (*S*)-[^18^F]OF-Me-NB1 over time in the same monkey. At 30 min after tracer injection, parent (*R*)-[^18^F]OF-Me-NB1 represented about 30% of the total plasma activity in the baseline scans, which further decreased to 20% at 90 min. Parent fraction of (*S*)-[^18^F]OF-Me-NB1 was lower than the (*R*)-enantiomer, at ~ 20% at 30 min and ~ 10% at 90 min post-injection. Pretreatment with the NMDA GluN2B blocking drug Co101,244 appeared to accelerate the decrease in parent fraction. On the other hand, pretreatment with FTC-146 had little effect. Plasma free fraction was 1.2 ± 0.2% (*n* = 6) for (*R*)-[^18^F]OF-Me-NB1 and 1.8% (*n* = 1) for (*S*)-[^18^F]OF-Me-NB1.

#### **Brain Analysis**

**Fig. 4:**
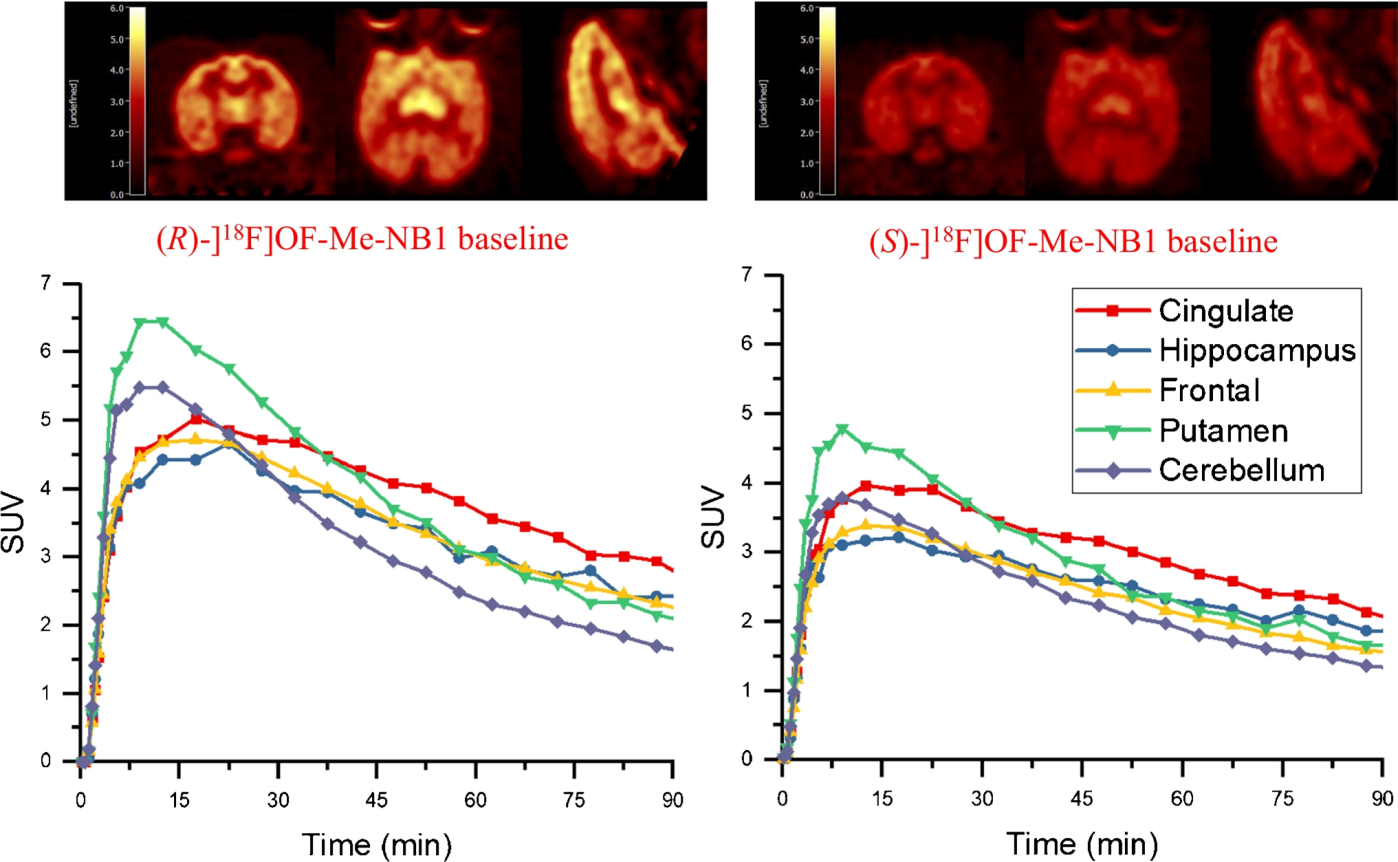
PET images of (*R*)- and (*S*)-[^18^F]OF-Me-NB1, summed from 30 to 45 min, in the same monkey brain (top), and the corresponding time-activity curves (bottom)

In the monkey brain, (*R*)-[^18^F]OF-Me-NB1 displayed good uptake and a heterogeneous distribution pattern (Fig. 4, top left). (*S*)-[^18^F]OF-Me-NB1 also displayed good brain uptake and a more homogenous distribution pattern, consistent with the results from rodents [23] (Fig. 4, top right).

The regional time-activity curves (TACs) from the baseline scans of (*R*)- and (*S*)-[^18^F]OF-Me-NB1 are also shown in Fig. 4. (*R*)-[^18^F]OF-Me-NB1 displayed fast and high brain uptake, as well as fast clearance. Peak uptake SUV of 4.5–6.5 across brain regions was reached within 20 min after tracer injection. Higher activity concentrations were found in the cingulate cortex, medium in the frontal cortex, hippocampus, and putamen, and low in the cerebellum, which is consistent with the expression of GluN2B subunits in the brain. (*S*)-[^18^F]OF-Me-NB1 also displayed fast and high brain uptake, and fast clearance, with peak SUV of 3.0–4.5 across regions reached within 15 min. In comparison with (*R*)-[^18^F]OF-Me-NB1, the dynamic range of (*S*)-[^18^F]OF-Me-NB1 uptake across regions was narrower, indicating lower levels of specific binding. Therefore, subsequent experiments were performed with (*R*)-[^18^F]OF-Me-NB1 only.

**Fig. 5:**
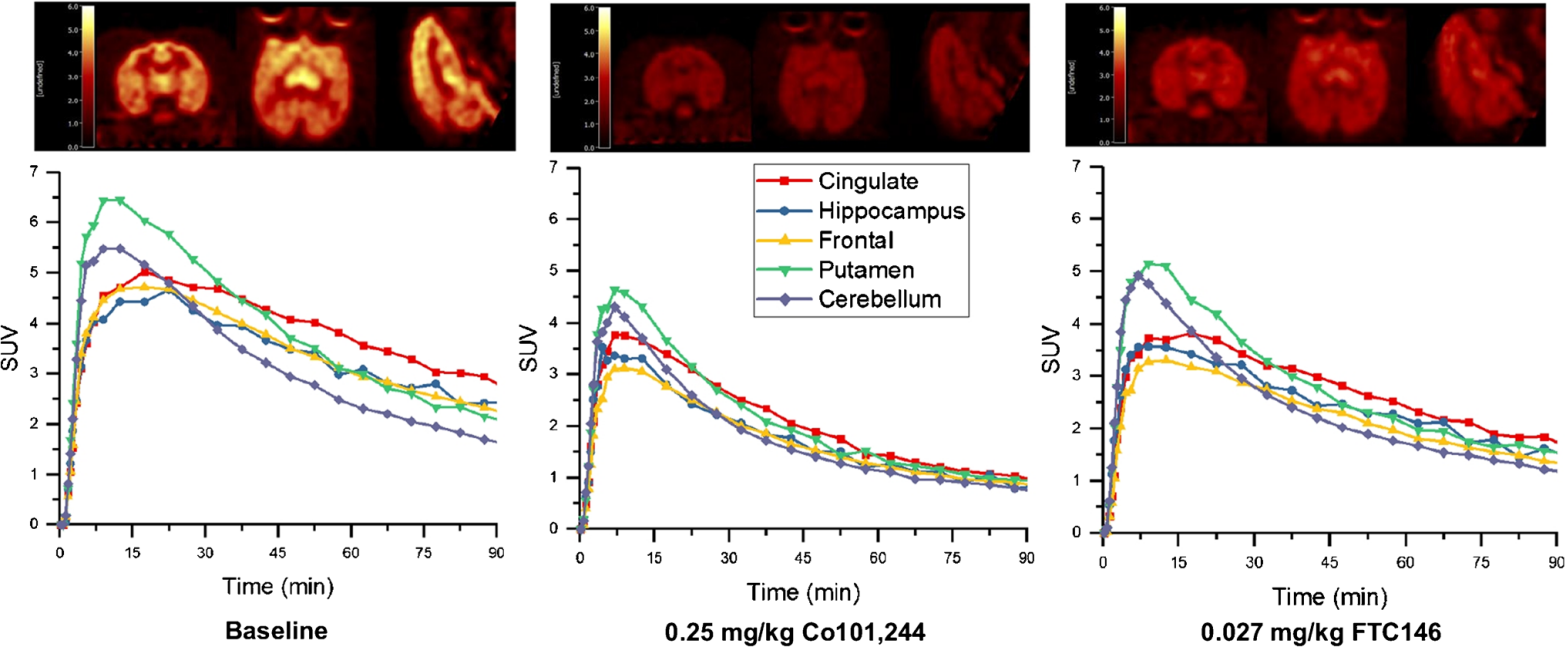
PET images of (*R*)-[^18^F]OF-Me-NB1 summed from 30 to 45 min, for the baseline scan (top left), and blocking scans with Co101,244 (0.25 mg/kg, top middle) and FTC-146 (0.027 mg/kg, top right), and the corresponding time-activity curves (bottom)

The blocking studies were conducted using the GluN2B antagonist Co101,244 and the selective sigma-1 receptor antagonist FTC-146 [25, 30, 31]. Pretreatment with Co101,244 (0.25 mg/kg) reduced the uptake of (*R*)-[^18^F]OF-Me-NB1 in all brain regions to nearly homogenous levels (Fig. 5, middle). Interestingly, FTC-146 also appeared to reduce the uptake of (*R*)-[^18^F]OF-Me-NB1 in all brain regions, although by a smaller magnitude compared to Co101,244 (Fig. 5, right). These trends were seen more clearly on the TACs. Co101,244 reduced activity concentrations in high and medium uptake brain regions to the same levels as those in the cerebellum and semiovale (white matter region) at the end of the scan, while FTC-146 induced partial reduction in radioactivity uptake across brain regions.

Time-activity curves were analyzed with the 1TC, 2TC, and MA1 methods to generate binding parameters using the metabolite-corrected plasma activity as input function. The 1TC and MA1 models showed better fits of the TACs than the 2TC model and values showed good agreement between the two. Thus, the MA1 was chosen as a suitable and reliable model for quantitative analysis. Regional *V*_T_ values derived from MA1 analysis (t* = 30 min) are listed in Table 1. In the baseline scans, regional *V*_T_ values followed the order of cingulate cortex > putamen > hippocampus > frontal cortex > thalamus > semiovale > cerebellum, which is consistent with the regional expression of GluN2B subunits in the brain. Blocking with either Co101,244 or FTC-146 led to decreased *V*_T_ values across all brain regions, with Co101,244 producing more pronounced decrease than FTC-146.

**Table 1:** MA1-derived distribution volume (*V*_T_) and non-displaceable binding potential (*BP*_ND_) of (*R*)–[^18^F]OF–Me–NB1 across brain regions in two rhesus monkeys (M1 and M2)

					*V*_*T*_ (mL/cm^3^)			
**Region of interest**	**Baseline**		**Co 101,244 (0.25 mg/kg)**		**FTC-146 (0.027 mg/kg)**	**FTC-146 (0.125 mg/kg)**	***BP***_**ND**_	
	M1	M2	M1	M2	M1	M2	M1	M2
**Cingulate cortex**	12.8	12.2	8.1	7.9	11.6	10.1	1.01	0.90
**Hippocampus**	11.0	10.3	6.9	7.3	10.5	9.2	0.73	0.60
**Frontal cortex**	10.7	10.3	6.8	7.4	9.4	9.2	0.68	0.60
**Thalamus**	9.4	9.6	6.8	6.8	9.4	8.6	0.48	0.50
**Semiovale**	9.1	9.5	8.6	8.0	9.5	8.4	0.43	0.48
**Cerebellum**	8.9	9.2	6.9	7.6	9.3	8.7	0.40	0.43

**Fig. 6:**
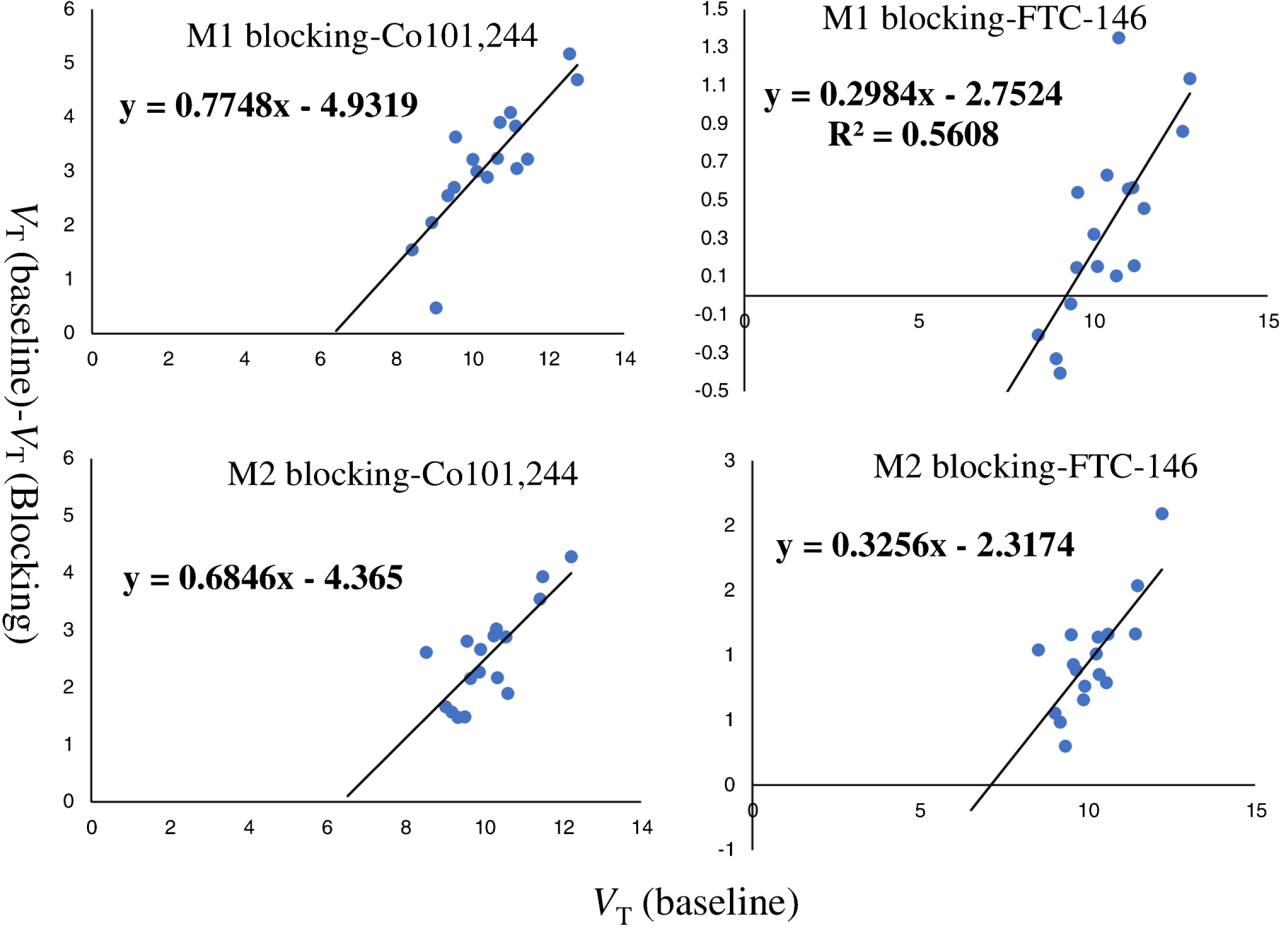
Receptor occupancy plots for (*R*)-[^18^F]OF-Me-NB1 blocking scans with Co101,244 (0.25 mg/kg) or FTC-146 (0.027 for M1 and 0.125 mg/kg for M2, respectively) in two different monkeys (M1 and M2)

Receptor occupancy by different blocking drugs was calculated using the occupancy plot (Fig. 6). In the male monkey (monkey 1), 0.25 mg/kg of Co101,244 resulted in 77% occupancy with V_*ND*_ of 6.4 mL/cm^3^, while a low dose of FTC-146 (0.027 mg/kg) gave about 30% occupancy. In the female monkey (monkey 2), Co101,244 at the same 0.25 mg/kg dose produced an occupancy of 68%, with the same V_*ND*_ of 6.4 mL/cm^3^. In this animal, a higher dose of FTC-146 (0.125 mg/kg) gave a similar level of occupancy, 33%, to the low dose in the male monkey. This indicates that the sigma-1 receptor antagonist, at both low (0.027 mg/kg) and high dose (0.125 mg/kg), produced a similar reduction in the binding of (*R*)-[^18^F]OF-Me-NB1 in the monkey brain [25].

Regional values of non-displaceable binding potential (*BP*_ND_) were calculated using the *V*_ND_ values derived from the occupancy plots with Co101,244 as blocking agent (Table 1). The rank order of regional *BP*_ND_ values was the same as that for *V*_T_, and consistent in both the male and female monkeys. These data demonstrate that in the monkey brain, (*R*)-[^18^F]OF-Me-NB1 specifically and selectively binds to the GluN2B receptor, and the specific binding can be reliably measured in monkey brain regions.

## Discussion

The GluN2B subunits of the NMDAR play an important role in psychiatric and neurological diseases, thus the development of diagnostic and therapeutic agents targeting this receptor will be valuable for the elucidation of its involvement in diseases. OF-Me-NB1, a close analog of Me-NB1, showed promising *in vitro* binding specificity to GluN2B subunits and selectivity over the sigma-1 receptor. The binding affinity (*K*_i_, nM) for GluN2B was 4 nM for (*R*)-OF-Me-NB1 and 37 nM for (*Rac*)-OF-Me-NB1. The *K*_i_ value for (*S*)-OF-Me-NB1 is not measured but expected to be one-fold higher than the (*R*)-enantiomer, based on the measured value for the racemic mixture. The selectivity of (*R*)-OF-Me-NB1 over sigma-1 receptor was good, with *K*_i_ > 100 nM (32 nM for (*Rac*)-OF-Me-NB1) [23]. (*R*)- and (*S*)-OF-Me-NB1 were previously labeled with either carbon-11 or fluorine-18 and evaluated in rodents. (*R*)-OF-Me-NB1 labeled with either carbon-11 or fluorine-18 exhibited distinct binding patterns consistent with GluN2B distribution and selectivity over sigma-1 receptors In rodent brain tissues, while (*S*)-[^11^C]OF-Me-NB1 showed a more homogenous distribution and binding to the sigma-1 receptor. The excellent binding properties of (*R*)-[^18^F]OF-Me-NB1 observed in rodent brains prompted us to fully evaluate this tracer in nonhuman primates with the aim to possibly advance it to human studies [23]. Although (*S*)-[^11^C]OF-Me-NB1 did not show promising attributes in rodents, we nonetheless decided to evaluate the ^18^F-labeled version given that the position of label potentially could make a difference in the performance characteristics of (*S*)-[^18^F]OF-Me-NB1 in the nonhuman primate brain. As such, both (*S*)- and (*R*)-[^18^F]OF-Me-NB1 were prepared using enantiopure acetyl protected boron pinacol ester precursors in a two-step, one-pot radiosynthetic approach. Both radioligands were obtained in > 98% radiochemical and enantiomeric purities. PET imaging studies in rhesus monkeys demonstrated that (*S*)-[^18^F]OF-Me-NB1 appeared to have less specific binding in the monkey brain, consistent with previously reported results in rodents [23]. In comparison, (*R*)-[^18^F]OF-Me-NB1 displayed higher specific binding that could be blocked by a GluN2B antagonist. (*R*)-[^18^F]OF-Me-NB1 exhibited a favorable metabolic profile and acceptable plasma-free fraction. All the radioactive metabolites were more polar than the parent tracer, thus less likely to cross the blood–brain barrier. The rapid brain uptake and clearance of (*R*)-[^18^F]OF-Me-NB1 in major brain regions indicates proper *in vivo* kinetics for future study in measuring GluN2B receptor changes. 1TC and MA1 analysis gave reliable and comparable *V*_T_ for both the male and female monkeys. Higher *V*_T_ values were found in the forebrain, including the cingulate cortex, putamen, and frontal cortex, and medium to low in white matter (centrum semiovale) and cerebellum. The value of regional *BP*_ND_, as a measure of specific binding signals, ranged from 1.0 in the cingulate cortex to 0.4 in the cerebellum in the male monkey, and 0.90 in the cingulate cortex to 0.43 in the cerebellum in the female monkey. These results indicate similar specific binding signals between sexes in nonhuman primates. The rank order of specific binding in different brain regions is consistent with the pattern of GluN2B expression in the monkey brain. The reduction of specific binding by the sigma-1 selective ligand FTC-146, albeit low, is intriguing. The low sigma-1 binding affinity of (*R*)-OF-Me-NB1 measured *in* *vitro* (*K*_i_ > 100 nM) suggests that this reduction is unlikely to reflect *in vivo* binding to the sigma-1 receptor by the radiotracer, but more likely due to the biological interaction between the sigma-1 and NMDA receptors. Such an explanation is supported by PET studies in rodents, where no differences were observed in the time-activity curves of (*R*)-[^18^F]OF-Me-NB1 in sigma-1 receptor knockout mice vs. wild-type animals [23].

## Conclusion

We have successfully synthesized and performed a comprehensive evaluation of (*R*)-[^18^F]OF-Me-NB1 as a selective radioligand for GluN2B in nonhuman primates. This novel radiotracer exhibits favorable metabolic, pharmacokinetics, and *in vivo* binding profiles. Given the promising *in vivo* characteristics of (*R*)-[^18^F]OF-Me-NB1 in the monkey brain, this radioligand holds promise for translating into humans.
